# RETRACTION: Lucidumol A, Purified Directly from *Ganoderma lucidum*, Exhibits Anticancer Effect and Cellular Inflammatory Response in Colorectal Cancer

**DOI:** 10.1155/ecam/9836935

**Published:** 2026-07-23

**Authors:** Evidence-Based Complementary and Alternative Medicine

RETRACTION: M.‐J. Shin, H.‐J. Chae, J. W. Lee, et al., “Lucidumol A, Purified Directly from *Ganoderma lucidum*, Exhibits Anticancer Effect and Cellular Inflammatory Response in Colorectal Cancer,” *Evidence-Based Complementary and Alternative Medicine* 2022, no. 1 (2022): 1–9, https://doi.org/10.1155/2022/7404493.

The above article, published online on 08 November 2022 in Wiley Online Library (https://wileyonlinelibrary.com), has been retracted by John Wiley & Sons Ltd.

The retraction has been agreed upon following an investigation into concerns raised by a third party and PubPeer comments [1] regarding image inconsistencies. Figure 3b cell migration assay 3.125 µM and 6.25 µM image sections contain areas of apparent overlap, which indicates that both sections originate from the same larger image. Additionally, the image representing 3.125 µM appears in a retracted earlier article by the authors under different conditions, Figure 6 Invasion 3.125 µM [2].

The authors were asked for an explanation and provided a response; however, their response was deemed insufficient for the image concerns present. The editors have lost confidence in the results; therefore, the article must be retracted.

The authors support the editor’s decision and agree with the retraction.
